# Editorial: Immune regulation and intervention in virus-related tumor development

**DOI:** 10.3389/fimmu.2023.1217980

**Published:** 2023-07-20

**Authors:** Qinglin Mo, Wei Zhu, Sha Wu

**Affiliations:** ^1^ Department of Immunology, School of Basic Medical Sciences, Microbiome Medicine Center, Guangzhou, China; ^2^ Department of Laboratory Medicine, Zhujiang Hospital, Southern Medical University, Guangzhou, China; ^3^ Key Laboratory of Proteomics of Guangdong Province, National Demonstration Center for Experimental Education of Basic Medical Sciences of China, Guangzhou, China; ^4^ State Key Laboratory of Organ Failure Research, Guangdong Provincial Key Laboratory of Viral Hepatitis Research, Department of Infectious Diseases, Nanfang Hospital, Southern Medical University, Guangzhou, China

**Keywords:** virus-related cancers, virus, tumor, immune regulation, immune intervention

Persistent infection with oncogenic viruses compromises the recognition, activation, and elimination abilities of cytotoxic immune cells, leading to exhaustion, anergy, senescence, activation-induced death, and apoptosis (1). Incompetence of the immunoprotective microenvironment facilitates the transformation of infection into cancer

Chronic infection dysfunctionalizes local and systemic immunity and facilitates primary carcinogenesis and secondary metastasis. Min et al. concluded that chronic HBV infection might cause the occurrence of extrahepatic tumors such as gastrointestinal tumors, and the poor prognosis of patients upon immune microenvironment change (immune-suppressive cytokine infiltration) and epigenetic modification by molecular signaling pathways and serum biomarkers such as hepatitis B virus X (HBx) protein. Zhao et al. showed that the existence of an oncogenic virus impacted the systemic anti-tumor effect, resulting in the interruption of chemotherapy and tumor development.

Stromal cells in a tumor microenvironment also orchestrate tumor progression. Li C. et al. found that cancer-associated fibroblasts (CAF) and C1QA^+^MRC1^high^ macrophages might be involved in lymph node metastasis of HPV-related cervical cancers (CC). As the main components in a solid tumor, stromal cells determine the antitumor immunity and tumor progression in the course of switching from anti-tumor effectors to pro-tumor effectors.

Immunotherapies including therapeutic vaccination, adoptive T cell therapy (ACT), and immune checkpoint blockade (ICB) have rapidly advanced and have become promising selections for virus-related tumors treatment (2). Li W. et al. and Yu et al. summarized the advancements in immunotherapies of virus-related tumors, among which ACT and ICB displayed promising prospects and were validated through clinical studies. Moreover, tumor-infiltrating lymphocyte (TIL) has been approved by the FDA for clinical usage in HPV-related CC. A considerable number of immune checkpoint inhibitors have also been licensed for use in EBV-related NPC by the FDA (Li W. et al.; Yu et al.).

Recognition is the initial step for cytotoxic immune cells to kill/remove virus-related tumor cells. However, recognition highly depends on the virus heterogenicity and deficiency of the broadly expressed target that can be recognized by cytotoxic immune cells. Srivastava et al. observed the previously unreported peptide GSLpqehivQK (POL606-616) that could be conserved across multiple HBVs with different genotypes and was found to present in most HLA-A11+ patients. This new peptide could be inserted into regions of the HBV genome and eventually presented on the surface of HBV-associated HCC. It may be a novel target that cytotoxic T-cells can recognize to mediate adaptive immunity.

An oncogenic virus plays the role of both the trigger and executor in tumor progression, and its virus epitopes could be specific and safe targets for immunotherapy, avoiding off-target effects. However, immunotherapy for virus-related tumors still faces some challenges, including re-infection, immunosuppressive microenvironment, financial cost, and immune-related adverse effects (irAEs).

## Author contributions

SW and WZ made the structure of the editorial, and QM finished the manuscript. All authors contributed to the article and approved the submitted version.
